# Disease-Associated miRNA-mRNA Networks in Oral Lichen Planus

**DOI:** 10.1371/journal.pone.0063015

**Published:** 2013-05-27

**Authors:** Volker Gassling, Jochen Hampe, Yahya Açil, Jan Hinrich Braesen, Jörg Wiltfang, Robert Häsler

**Affiliations:** 1 Department of Oral and Maxillofacial Surgery, University Hospital of Schleswig-Holstein, Campus Kiel, Kiel, Germany; 2 Department of Internal Medicine I, University Hospital of Schleswig-Holstein, Campus Kiel, Kiel, Germany; 3 Institute of Clinical Molecular Biology, University Hospital of Schleswig-Holstein, Campus Kiel, Kiel, Germany; 4 Department of Pathology, University Hospital of Schleswig-Holstein, Campus Kiel, Kiel, Germany; East Carolina University, United States of America

## Abstract

**Background:**

A large number of pathophysiological mechanisms are regulated by microRNAs (miRNAs), which represent a new class of posttranscriptional regulators of gene expression. To date, little is known about their role in oral lichen planus (OLP), a chronic inflammatory mucocutaneous disease of unknown etiology which is being discussed as a potentially premalignant condition of oropharyngeal cancer. The aim of the present investigation was to assess the pathophysiological impact of miRNAs and to determine regulatory miRNA networks which are directly linked to potentially disease-associated target transcripts in OLP.

**Methods:**

Native tissue samples were collected from the oral mucosa of seven patients with OLP. The control group was composed of native tissue from elective oral surgery. The mRNA profiling was performed using the Affymetrix Human Gene 1.0 ST Array while miRNA profiling was performed using the microRNA Galaxy Array. Subsequent validation of initial results was carried out using TaqMan real time PCR.

**Results:**

We identified 24 differentially regulated miRNA and 2,694 regulated transcripts. Linking the miRNAs to their potential targets we found 11 potential miRNA-mRNA pairs, of which several are functionally related to premalignant as well as to inflammatory events.

**Conclusions:**

Our data shows miRNA associated with transcripts which are regulated when comparing OLP patients with healthy control individuals. This suggests that miRNAs may potentially regulate disease-relevant transcripts, proposing the concept of therapeutic interventions based on miRNAs.

## Introduction

Oral lichen planus (OLP) is a chronic T-cell-mediated autoimmune disease of unknown etiology with higher incidences in females [1]. OLP is currently being discussed as a potential premalignant status, while controversial results do not allow final conclusions to be drawn [2], [3]. In this context, in 1997 the WHO classified OLP and other diseases (e.g. syphilis, oral submucous fibrosis, xeroderma pigmentosa, and discoid lupus erythematosus) as precancerous conditions. These are defined as: “A generalized state associated with a significantly increased risk of cancer development”. The only common and characteristically histological features of these diseases are increased mitotic activity, epithelial atrophy and decreased epithelial repair mechanisms. In contrast to OLP, leukoplakia and erythroleukoplakia, which are classified as precancerous lesions, are defined as “A morphologically altered tissue in which cancer is more likely to occur than in its apparently normal counterpart” [4], [5]. Due to the lack of characteristically histological criteria for precancerous conditions, the WHO classification is based on observations that cancer may develop in diseases such as e.g. OLP after a certain period of time rather than being supported by the occurrence of molecular alterations as in precancerous lesions, e.g. leukoplakia [6].

A worldwide epidemiologic comparison of new cancer cases has revealed that oropharyngeal cancers are amongst the 10 most common cancer types with an incidence of 403,000 cases in 2002, whereby two thirds of all those cases occurred in developing countries [7]. To date, the management of oropharyngeal cancer has consisted of a combination of surgery, radiotherapy, and chemotherapy. Despite considerable effort in these therapies, the 5-year survival rate even in developed countries has not improved significantly and remains in Germany for example at about 44% for men and 59% for women [8]. This illustrates the importance of further unraveling the etiopathogenic mechanisms of oropharyngeal cancer and the underlying malignant transformation of precancerous tissue to cancer tissue finally leading to the development of new therapeutic approaches.

In this context, micro-RNAs (miRNAs), a group of recently identified molecules which are being discussed as new transcription factors, may play a new role: They are phylogenetically conserved, non-protein-coding, small single stranded RNAs. After maturation, miRNAs are incorporated into an RNA-induced silencing complex (RISC) which regulates protein expression by binding to complementary sequences in 3′UTR of specific target mRNAs [9]. Until now, our knowledge of their function in apoptosis, development, differentiation, metabolism, and proliferation by regulating the expression of different signaling molecules has grown rapidly [10]. A few years ago, about 700 miRNA sequences had been identified but it was estimated that more than 1,000 exist in the human genome [11]. Now, seven years later, about 1,500 miRNAs are listed in the miRBase (www.mirbase.org), while next generation sequencing data suggests that the miRNA world is substantially larger. Estimations predict that at least 10% [12] to 30% [13] of all gene transcripts are regulated by miRNAs. This suggests that each miRNA can control a large number of target mRNAs and that each mRNA can be controlled by many miRNAs.

Due to their regulatory nature in physiology, miRNAs represent a mechanism of high relevance for health and diseases, including cancer. It has been shown that in human cancers global alterations in miRNA expression are a common phenomenon [14]. There is evidence that miRNAs play a potential role in cancerogenesis of head and neck squamous cell carcinoma (HNSCC) [15], and recent findings suggest that there is a particular miRNA signature associated with the progression of premalignant lesions, e.g. the transformation from oral leukoplakia to oral carcinoma [16]. The critical role of several miRNAs in this malignant transformation has also been assessed, highlighting the relevance of over-expression of miR-21, miR-181b, and miR-345 in this process. In the same line, miR-21 has been shown to be associated with cancer development and cell proliferation [17]. In general, it has been hypothesized that the majority of such alterations occurs before the development of malignancy [18].

In the light of the regulatory properties of miRNAs and due to previous studies, which have documented the impact of miRNAs on tumor development and progression, we aimed to assess the potential relevance of miRNA patterns and their possible interaction partners on mRNA levels in primary tissue from OLP patients. Combining genome-wide quantification of miRNAs and mRNAs levels and comparing OLP patients to healthy individuals allows for an identification of disease-associated events, enabling the creation of a genome-wide map and the identification of candidate-pairs of miRNA-mRNA interactions, which can serve as a starting point for the development of new diagnostic and therapeutic approaches.

## Materials and Methods

### Patient recruitment and ethics statement

Native tissue samples were collected from consultation hours for oral mucosa at the Department of Cranio-Maxillofacial Surgery, University Hospital Schleswig-Holstein, Campus Kiel, Kiel, Germany. Exploratory clinical examinations of 117 patients were performed within the context of the study. Seven patients with reticular OLP in anamnesis and typical clinical features (see details below) where attended and specimens were taken at regular intervals. This concept was to ensure that only tissue samples from OLP and not oral lichenoid reactions were included for further analysis. One half of the specimens was examined at the pathology department to verify the diagnosis of OLP by specific histopathological criteria (see details below). The other half was stored in liquid nitrogen for further analysis. Only samples which fulfilled the characteristic clinical and histopathological criteria were used for analysis. The control group was composed of native tissue from elective oral surgery whereby histopathology examination excluded any diseases of the oral mucosa. The study was approved by the ethics board of the Christian-Albrechts-University of Kiel, Germany (Ref: D 426/08). All the participants were informed by a patient information sheet about the intention of the study and additional anonymous genetic analysis of tissue samples. They gave their written informed consent according to the Helsinki convention.

### Inclusion criteria

According to the suggestions of van der Meij et al [19], we set the following clinical and histological inclusion criteria based on the World Health Organization diagnostic criteria for OLP and oral lichenoid lesions (OLL) [20].

Clinically active OLP was defined by the presence of bilateral, mostly symmetrical lesions, by the presence of a lacelike network of slightly raised gray-white lines in a reticular pattern and by erosive, atrophic, bullous, and plaque-type lesions (accepted as a subtype only in the presence of reticular lesions elsewhere in the oral mucosa).

Histological inclusion criteria were the presence of well-defined bandlike zones of cellular infiltration confined to the superficial part of the connective tissue, consisting mainly of lymphocytes, signs of “liquefactive degeneration” in the basal cell layer and the absence of epithelial dysplasia.

Clinical as well as histopathological criteria had to be fulfilled for final OLP diagnosis. In this article, the term OLL describes i) clinically typical OLP which is only histopathologically compatible with OLP, ii) histopathologically typical OLP which is only clinically compatible with OLP and iii) conditions clinically compatible with OLP which are histopathologically compatible with OLP.

### RNA extraction, mRNA/miRNA profiling and validation of screening results

Total RNA including miRNA was extracted from biopsies homogenized under liquid nitrogen to avoid RNA degradation using miRVana (Ambion, Life Technologies, Carlsbad, CA) and further processed as previously described [21]. The quality control of the RNA was carried out using an Agilent Bioanalyzer (Agilent, Böblingen, Germany). mRNA profiling was performed using the Affymetrix Human Gene 1.0 ST Array (Affymetrix, Santa Clara, CA) while miRNA profiling was performed using the microRNA Galaxy Array (Affymetrix, Santa Clara, CA). Both array types were processed according to the manufacturers' guidelines. Validation of initial results via real-time PCR (TaqMan) was carried out according to the manufacturers guidelines (Applied Biosystems) employing a 7900HT Real-Time PCR System. Transcript levels were calculated relative to beta-actin (for mRNA) and small nucleolar RNA, C/D box 47 (SNORD47, for miRNA) using the standard-curve method [22]. Each measurement was performed in technical triplicates. Validation results were correlated with initial screening results by calculating Spearman's rank correlation coefficient. This observed correlation was then compared to (k = 10.000) permuted correlations, where validation results were associated to randomly picked screening results based on a Westfall and Young permutation [23]. Finally, the permutation results were used to estimate the probability of the observed correlation occurring by chance.

### Data analysis

Data were normalized using RMA (R, Bioconductor). The resulting data was submitted according to MIAME standards to Gene Expression Omnibus (GEO, URL: http://www.ncbi.nlm.nih.gov/geo; super-series: GSE38617; miRNA data: GSE38615; mRNA data: GSE38616). Differences between experimental groups were assessed by calculating the rank-sum difference, while transcripts with the theoretical maximal rank sum difference were considered as distinctly expressed. Signed fold changes were calculated based on the ratios of the relative expression values (lichen planus patients vs. healthy individuals). P-values were calculated using the Mann-Whitney U-test.

To compensate for the small sample sizes and the potential confounding factor introduced by this, identified mRNA transcripts and miRNAs were subjected to a cluster analysis and to principal component analysis (PCA) to verify their ability to separate lichen planus patients from healthy individuals (see below).

Cluster analysis was carried out using TIBCO SPOTFIRE IBD (Tibco, Palo Alto, CA), using an unweighted average with correlation as a similarity measure. The PCA was performed using TIBCO SPOTFIRE IBD (Tibco, Palo Alto, CA) based on z-score-normalized expression values. Analysis of the biological processes was conducted as previously published [24], and gene ontology terms (category: biological processes) were retrieved from the Gene Ontology Consortium (www.geneontology.org). Categorical associations of biological processes to transcripts were analyzed by counting occurrences of terms for each group of transcripts or miRNAs individually. Target transcripts of miRNAs were predicted using an algorithm which takes into account the secondary as well as the tertiary structure of both the miRNA and its target mRNA as previously published [25].

## Results

In the genome-wide mRNA screen, in which a total number of 33,297 transcripts were quantified, 26,206 were categorized as present in the tissue investigated. Of these, 2,694 were regulated between OLP patients and healthy individuals (p-value ≤ 0.05; 492 upregulated; 2202 downregulated), while 57 of those were categorized as distinctly expressed. Distinctly expressed transcripts are defined as transcripts where values from OLP patients show no overlap with healthy individuals (all relative expression values of one group are higher than the relative expression values of the other group). From those 57, 44 transcripts were associated with characterized genes (listed in Table S1).

In the genome-wide miRNA screen, which targeted 7,656 different miRNAs and other non-coding RNAs, 266 were categorized as present in the tissue investigated and of human origin. Of those, 24 were identified as differentially expressed (p-value ≤ 0.05), of which 16 were associated with miRNAs (listed in Table S2).

**Figure 1:**
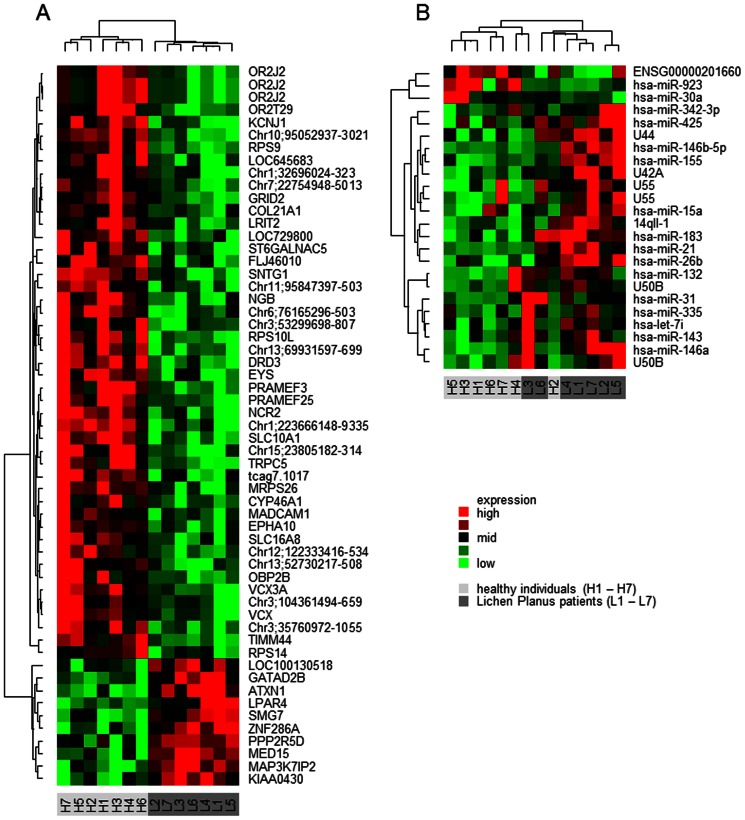
Lichen planus mRNA/miRNA expression patterns. Cluster analysis of mRNAs (A) and miRNAs (B). Expressed features are represented in rows while patients are represented in columns. The heat map is color-coded according to the relative expression. Vertical dendograms display the similarity between the expressed features and horizontal dendograms display the similarities between the patients. Features are labeled with gene symbols (or with public references, if no gene symbols are available or with the genomic alignment where no public references are available) or with miRNA names, respectively. Multiple appearances of expressed features (e.g. OR2J2) are a result of one gene being represented by multiple transcripts which were all differentially expressed. Samples are labeled with H1–H7 for healthy individuals and L1–L7 for Lichen Planus patients.

Cluster analysis of gene transcripts, based on 57 differentially expressed transcripts (Figure 1A) resulted in the clear separation of OLP patients and healthy individuals, displaying up-regulated as well as down-regulated transcripts. Similarly, the cluster analysis of 24 selected miRNAs resulted in a separation of OLP patients from healthy individuals, while one healthy individual clustered with the OLP patients (Figure 1B).

**Figure 2:**
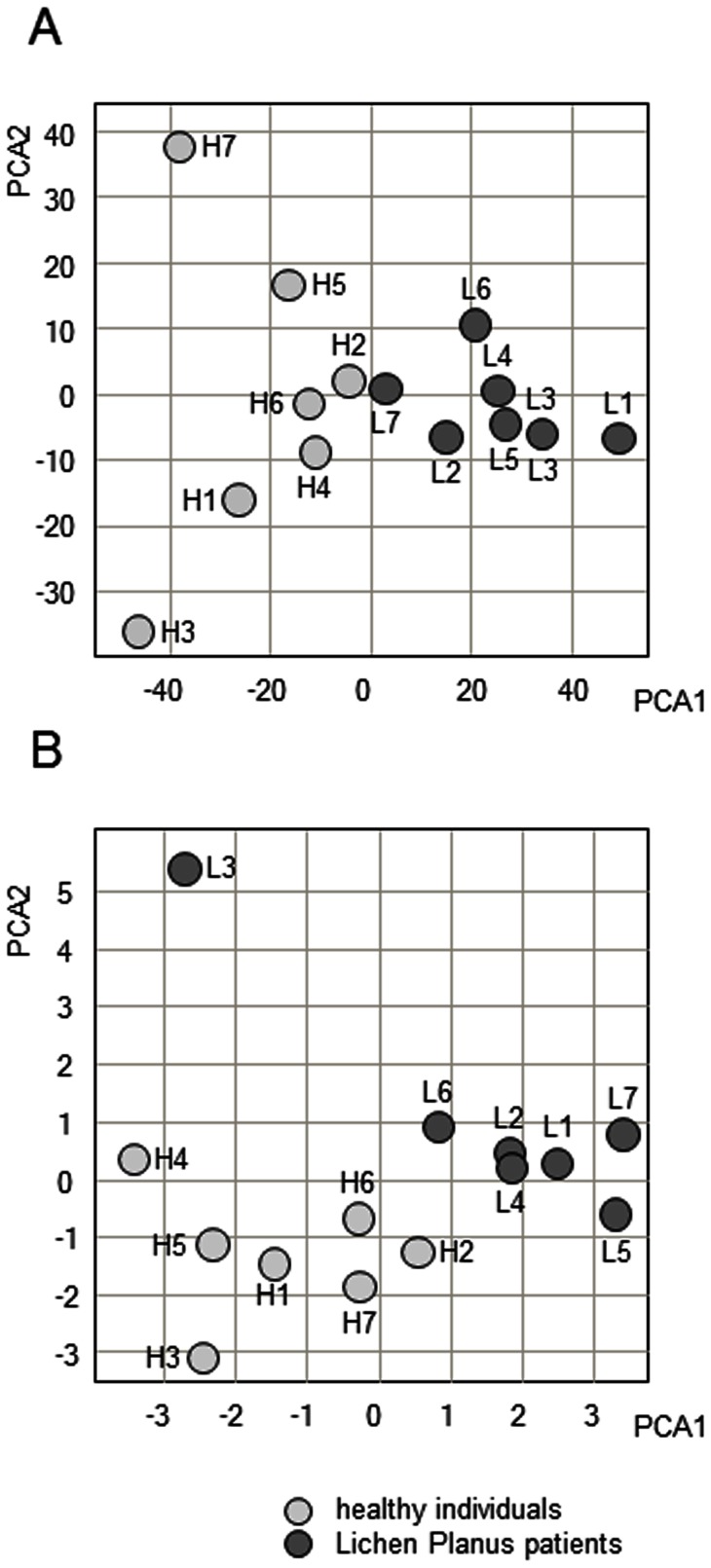
Principal component analysis of lichen planus patterns. The two strongest components (X-axis: PCA1, Y-axis: PCA2) are shown for a PCA based on differentially expressed transcripts (A) and based on differentially expressed miRNAs (B). Each data point on the plot represents one healthy individual (light gray, labeled H1–H7) or one Lichen Planus patient (dark gray, labeled L1–L7).

In concordance with the cluster analysis, the PCA based on the same genes showed that the phenotype is the component of the largest part of the variation (PCA1) for both mRNA (Figure 2A) and miRNA (Figure 2B). By including a second dimension (PCA2), both phenotypes could be separated with no outliers.

**Figure 3:**
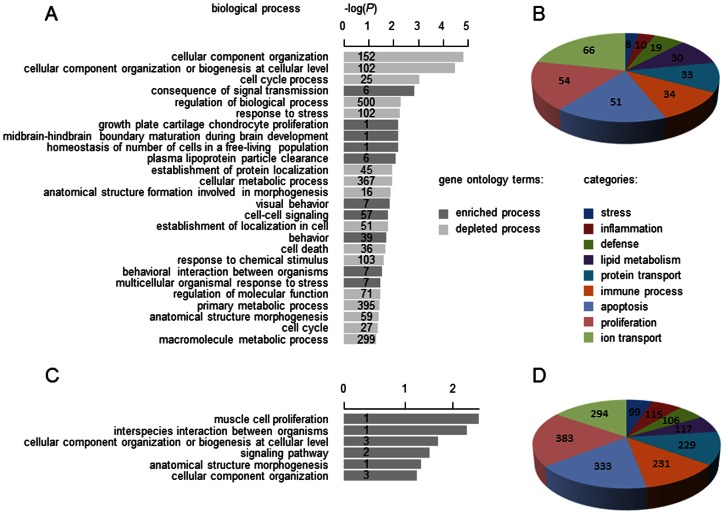
Enriched and depleted biological processes in lichen planus. Biological processes (retrieved from the Gene Ontology Consortium) associated with differentially expressed transcripts (A) or grouped into most prominent categories (B). Level 1 terms (e.g. “system process” or “signal transduction”) were omitted. Each regulated item was associated to its biological processes using Gene Ontology, the resulting hits were summarized for each category of biological processes, illustrated by individual elements in the pie-chart. Similarly, biological processes (C) and categories (D) are shown for differentially expressed miRNAs. Numbers next to biological processes and categories indicate the number of transcripts or miRNAs observed.

The gene ontology analysis based on the 2,694 differentially regulated transcripts identified various processes as significantly enriched or depleted. A large number of transcripts in this analysis were associated with processes in the context of cell growth, proliferation and the reorganization of tissue (Figure 3A). Similarly, such processes were observed when analyzing the terms associated with the targets of the miRNAs. In contrast to the transcripts, the miRNA targets showed only a small number (1–3 vs. 7–500) of transcripts per term (Figure 3C).

A categorical approach of assessing with which terms both regulated mRNAs and miRNA-targets are associated identified a similar picture for both: proliferation, apoptosis, ion transport and the immune process represented the major categories in regulated transcripts and predicted targets of the differentially expressed miRNAs (Figure 3B and D).

**Figure 4:**
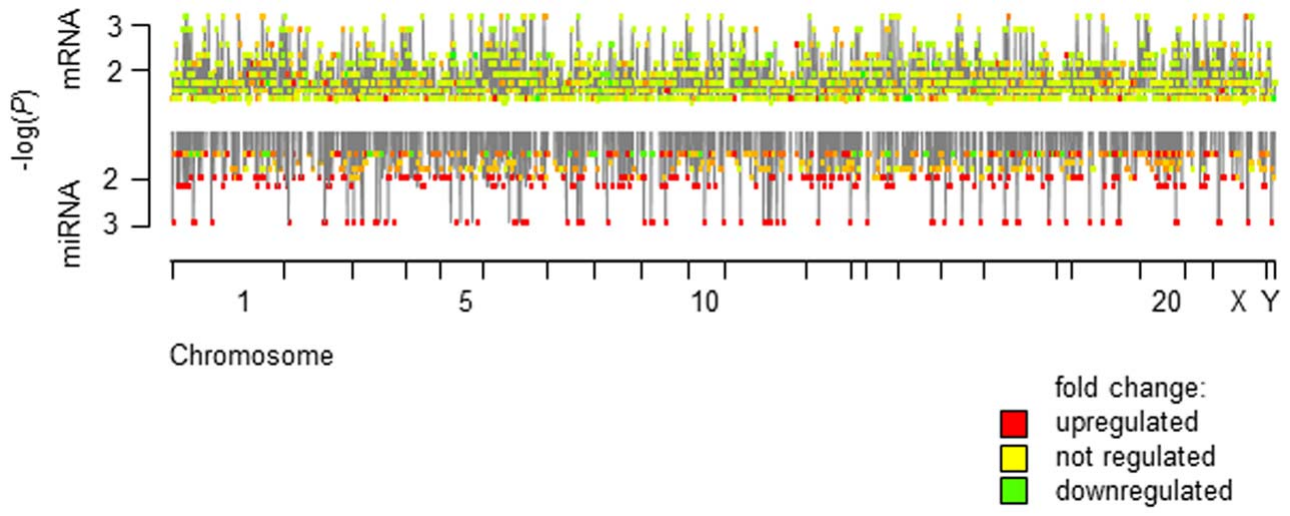
Genomic map of regulated transcripts and miRNAs in lichen planus. A non-linear display of the genomic location is plotted on the X-axis, while the length of each chromosome plotted is determined by the amount of features identified in the chromosome. The significance is plotted as –log(P) on the y-axis for regulated transcripts and miRNAs in the corresponding genomic location. Data points are color-coded by fold change.

An alignment of miRNAs and their targets on the genome to identify potential regulatory hotspots did not reveal any regions of special interest (Figure 4).

**Figure 5:**
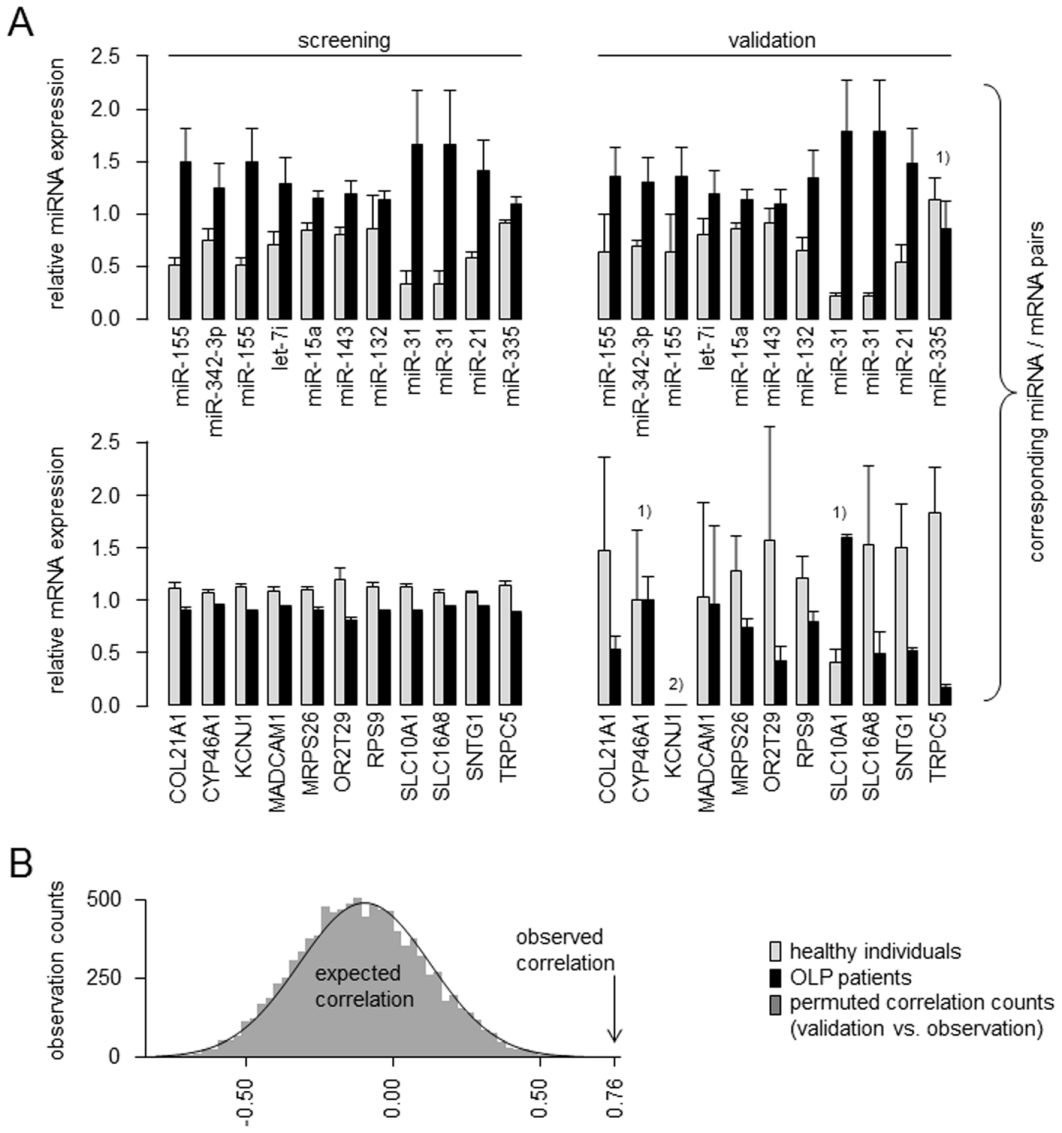
Selected candidate transcripts under potential miRNA control. 5A) Regulated miRNAs (first row) are displayed with their predicted target mRNA (second row), in which the miRNAs were up-regulated when comparing lichen planus patients with healthy individuals, and their predicted target transcripts were down-regulated (left panel: initial screening; right panel: validation via real-time PCR). The y-axis displays the relative expression normalized to beta-actin (for mRNAs) or to small nucleolar RNA, C/D box 47 (SNORD47, for miRNAs). Error bars represent the standard error of the mean. 5B) The observed correlation between screening and validation results (rho = 0.765) is compared to expected correlations (based on a k = 10.000 Westfall and Young permutation). ^1)^ Transcripts, where real-time PCR validation does not support initial screening results ^2)^ KCNJ1 did not result in detectable signals in the validation step.

Finally, linking differentially expressed miRNAs to their potential differentially expressed target using the algorithm published by Kertesz et al [25] identified a total of 11 potential miRNA-mRNA pairs: COL21A1 (collagen, type XXI, alpha 1; targeted by hsa-miR-155), CYP46A1 (cytochrome P450, family 46, subfamily A, polypeptide 1; targeted by hsa-miR-342-3p), KCNJ1 (potassium inwardly-rectifying channel, subfamily J, member 1; targeted by hsa-miR-155), MADCAM1 (mucosal vascular addressin cell adhesion molecule 1; targeted by hsa-let-7i), MRPS26 (mitochondrial ribosomal protein S26; targeted by hsa-miR-15a), OR2T29 (olfactory receptor, family 2, subfamily T, member 29; targeted by hsa-miR-143), RPS9 (ribosomal protein S9; targeted by hsa-miR-132), SLC10A1 (solute carrier family 10 (sodium/bile acid cotransporter family), member 1; targeted by hsa-miR-31), SLC16A8 (solute carrier family 16, member 8 (monocarboxylic acid transporter 3); targeted by hsa-miR-31), SNTG1 (syntrophin, gamma 1; targeted by hsa-miR-21) and TRPC5 (transient receptor potential cation channel, subfamily C, member 5; targeted by hsa-miR-335). All pairs are characterized by differential expression where the regulation of the mRNA is in opposite direction to the regulation of the miRNA. MiRTarBase lists all of these pairs as currently not experimentally validated [26]. However, the number of validated miRNA-target interactions is still very low (status March 2013: 317 miRNAs linked to 1960 mRNAs), indicating that current approaches should not be limited to validated miRNA-target pairs. The selected candidate pairs were subjected to verification via real-time PCR (TaqMan), resulting in 18 of 22 signals showing the same regulation as in the initial screening (Figure 5A). The correlation between screening and validation results was calculated to be at 0.765, which is defined to be a “strong correlation”. A Westfall and Young permutation (k = 10.000) resulted in a probability of less than 0.01% that the observed correlation could have occurred by chance (Figure 5B).

## Discussion

The etiology of OLP and its potential to represent a premalignant phenotype is still poorly understood [2], [3], [27]. Recently, it has been suggested that miRNAs play a role in HNSCC cancerogenesis [28]. In addition, it has been shown that miRNA may be used to predict the risk of Oral Premalignant Lesions [29]. Combining genome-wide miRNA profiles with genome-wide mRNA profiles represents a powerful tool in the identification of regulatory networks, as has been demonstrated in previous studies [30]–[32]. The findings of the presented study illustrate that miRNAs may represent an important controlling element, showing regulatory effects on their target transcripts and thus contribute to OLP susceptibility, manifestation and progression.

### Monitoring disease-associated events in primary tissue

Primary tissue from clinical samples with its many sources of heterogeneity (e.g. in cell composition) can result in a number of confounding factors in transcriptome profiling as well as in miRNA profiling. In this context, it is important to note that several studies have shown that primary tissue can serve as a powerful tool in the identification of complex pathophysiological mechanisms, where monitoring different cell types may play an important role [33], [34]. Similarly, a changed cell composition might reflect an important event in OLP manifestation or progression. In this context, it has been shown that cultured tumor cell lines are inappropriate for miRNA biomarker identification in HNSCC, whereas primary head-neck tissue provides a better picture of the disease's status [35]. The non-parametric methods employed allow for compensation of the high variation encountered in clinical settings. Interestingly, the variations between patients observed in this study were mostly found on the miRNA level, not on the transcriptome level. The observation that some healthy individuals cluster next to lichen planus patients not only illustrates this heterogeneity in a clinical setting, but also reflects the dynamic range of molecular patterns which can be attributed to a healthy phenotype. In addition, variations between patients in disease-associated mRNAs and miRNAs may not only be the result of different genetic backgrounds, but also the result of different environmental influences. Variation in gene expression is partially attributed to the amount of genetic and non-genetic (e.g. environmental) control [36]. In the context of OLP, several studies have indicated that a combination of triggering factors, such as genetic and environmental factors, may be required for disease manifestation [37]. Naturally, the setup employed does not allow monitoring transcriptional repression, therefore the observed disease-associated mRNAs and miRNAs may reflect only part of the regulatory miRNA-network found in OLP.

### Biological processes associated with regulated miRNAs and mRNAs

This expected environmental influence is also reflected by the biological processes associated with the regulated transcripts: response to stress is one of the most prominent terms showing a significant enrichment. Many other biological processes associated with regulated transcripts however illustrate the potentially premalignant nature of the tissue investigated: e.g. cell cycle, cellular density control and cell death, to name but a few. Interestingly, when using a categorical approach, high similarities between processes associated with regulated transcripts and processes associated with predicted targets of regulated miRNA have been observed, further supporting the hypothesis of two closely interacting elements in the pathophysiology of OLP.

### Disease-associated miRNA/mRNA pairs

A recent study has documented the impact of genetic variation on miRNAs, showing that miRNA-related variants are associated with the development of premalignant oral lesions [29]. Our approach does not allow the creation of a direct link to those variants, but it further illustrates the role of miRNAs in the interplay between the susceptibility, manifestation and disease progression of OLP. Selecting both disease-associated miRNAs and disease-associated mRNAs to suggest a functional link has been shown as a valid experimental setup: miRNAs regulated in response to inflammatory stimuli-controlled and inflammation-associated transcripts [38]. Similarly, OLP-associated miRNAs were able to regulate OLP-relevant transcripts. On a genome-wide scale, it is interesting to note that our study does not provide evidence of structural variation, e.g. massive deletions resulting in no expression of an entire genomic region. In contrast to previously reported operon-like gene structures, that are transcribed from a common promoter [39], [40], we did not find such patterns, nor could we observe any genomic hotspots. Currently, there are no studies providing experimental support for the hypothesis of this operonic organization being functionally relevant.

### Potential clinical relevance of individual miRNA/mRNA pairs

Several of the transcripts discussed in the present study have been shown to be involved in cancer development. In particular, COL21A1, which we observed to be down-regulated and potentially controlled by miR-155 is known to be important for cell plasticity and extracellular matrix remodeling and has been reported to be involved in tumor cell invasion and metastasis [41]. Another factor contributing to tumor progression is an inappropriate immune response. This is functionally linked to the decrease in mucosal homing alpha4beta7+ T-cells in tumor tissue, while this is probably due to the correlated decrease in MADCAM-1 positive blood vessels in tumor mucosa [42], which was also shown to decrease in the presented study and potentially controlled by miR let 7i. In contrast to our findings, a previous study showed that the inhibition of RPS9 expression may be one efficient way to inhibit rapidly proliferating tumor cells [43]. Transient receptor potential cation (TRPC) channels have been found to be involved in the abnormal proliferation, differentiation, and growth of cancer cells [44]. Deregulation of such transcripts may indicate both pathophysiological mechanisms of tumor progression and endogenous tumor defense mechanisms aiming at keeping the tumor growth under control. The essential role of miRNAs in cancer development has been suggested by several studies. A recently published meta-analysis of 13 published miRNA profiling studies identified 67 commonly deregulated miRNAs in head and neck cancer. They reported the up-regulation of miR-21, miR-155, miR-130b, miR-223 and miR-31, which is in concordance with our findings [45]. In contrast, in an experimental study of oral squamous cell carcinoma in Syrian hamsters, miR-155 and miR-143 were down-regulated, whereby miR-21 was up-regulated [46], yet it is unclear how to compare this model system to the human *ex-vivo* data presented here. Furthermore, in oral squamous cell carcinomas, an under-expression of miR-155, miR-let-7i, and miR-146a was found to characterize progression to metastatic tumors [47]. As these miRNAs were up-regulated in OLP, this further supports the hypothesis that different tumors exhibit different miRNA profiles. MiR-132, which targets the retinoblastoma tumor suppressor gene and leads to enhanced cell proliferation [48], has been shown to be over-expressed in tongue carcinomas [49], which was also found in our study. In addition, miR-31, miR-155 and miR-21 were up-regulated in HNSCC [45], [50], which is also in concordance with our observation. Remarkably, it has been shown that the plasma miR-31 in patients with oral squamous cell carcinomas was reduced after tumor resection suggesting that this marker is tumor-associated [51]. In contrast, blocking miR-31 expression reduced the growth of tumor xenografts [52]. The technical validation performed on all the presented 11 mRMA-miRNA pairs further supports our hypothesis of potential disease relevance.

Taken together, our genome-wide map of a regulatory miRNA/mRNA network which is in concordance with many previous studies as well as the identified miRNA/mRNA pairs represents a starting point for further studies: Assessing the direct interaction of regulated miRNAs and their suggested targets is one of the next steps required to broaden our understanding of OLP. Similarly, it would be interesting to see the impact of the identified candidates in i) larger cohorts of patients and ii) other premalignant disorders.

## Conclusions

Our study for the first time shows that miRNAs and their potential target transcripts may represent a regulatory network, controlling disease-associated processes in OLP. Keeping in mind that the study setup does not allow the identification of a causal effect, the results suggest that miRNAs and their modulation should be considered as targets for future therapeutic strategies.

## Supporting Information

Table S1 Transcripts, distinctly expressed between OLP patients and healthy individuals. All differentially expressed transcripts associated with a characterized gene are listed with their potential interacting miRNA. Fold changes are based on ratios (relative expression in OLP vs. relative expression in healthy individuals); p-values are based on a Mann-Whitney U-test.
(DOCX)

Table S2 miRNAs, differentially expressed between OLP patients and healthy individuals. All differentially expressed non-coding RNAs associated with a characterized miRNA are listed. Fold changes are based on ratios (relative expression in lichen planus patients vs. relative expression in healthy individuals); p-values are based on a Mann-Whitney U-test.
(DOCX)
