# Supplementary material for: Disease-Associated miRNA-mRNA Networks in Oral Lichen Planus
Source: PLoS One. 2013 May 27;8(5):e63015. doi: 10.1371/journal.pone.0063015 (PMC3664564; doi:10.1371/journal.pone.0063015)
Supplement: Table S1 — Transcripts, distinctly expressed between OLP patients and healthy individuals. All differentially expressed transcripts associated with a characterized gene are listed with their potential interacting miRNA. Fold changes are based on ratios (relative expression in OLP vs. relative expression in healthy individuals); p-values are based on a Mann-Whitney U-test. (DOCX) [file pone.0063015.s001.docx]

**Supplemental Table 1** Transcripts, distinctly expressed between OLP patients and healthy individuals. All differentially expressed transcripts associated with a characterized gene are listed with their potential interacting miRNA. Fold changes are based on ratios (relative expression in OLP vs. relative expression in healthy individuals); p-values are based on a Mann-Whitney U-test.

| **Gene symbol** | **Gene name** | **Targeted by** [hsa-miR-] | **Fold change** | **p-value** |
| --- | --- | --- | --- | --- |
| **Upregulated mRNA transcripts** | | | | |
| LPAR4 | lysophosphatidic acid receptor 4 | - | 1.57 | 0.00058 |
| ZNF286A | zinc finger protein 286A | - | 1.51 | 0.00058 |
| MAP3K7IP2 | mitogen-activated protein kinase kinase kinase 7 interacting protein 2 | - | 1.35 | 0.00058 |
| ATXN1 | ataxin 1 | - | 1.26 | 0.00058 |
| GATAD2B | GATA zinc finger domain containing 2B | - | 1.26 | 0.00058 |
| SMG7 | Smg-7 homolog, nonsense mediated mRNA decay factor (C. elegans) | - | 1.23 | 0.00058 |
| MED15 | mediator complex subunit 15 | - | 1.21 | 0.00058 |
| LOC100130518 | similar to hCG1773661 | - | 1.17 | 0.00058 |
| KIAA0430 | KIAA0430 | - | 1.14 | 0.00058 |
| PPP2R5D | protein phosphatase 2, regulatory subunit B', delta isoform | - | 1.10 | 0.00058 |
|  |  |  |  |  |
| **Downregulated mRNA transcripts** | | | | |
| ST6GALNAC5 | ST6 (alpha-N-acetyl-neuraminyl-2,3-beta-galactosyl-1,3)-N-acetylgalactosaminide alpha-2,6-sialyltransferase 5 |  | -1.18 | 0.00058 |
| EPHA10 | EPH receptor A10 | - | -1.17 | 0.00058 |
| FLJ46010 | FLJ46010 protein | - | -1.17 | 0.00058 |
| OBP2B | odorant binding protein 2B | - | -1.17 | 0.00058 |
| COL21A1 | collagen, type XXI, alpha 1 | 155 | -1.14 | 0.00058 |
| VCX | variable charge, X-linked | - | -1.14 | 0.00058 |
| NGB | Neuroglobin | - | -1.13 | 0.00058 |
| SNTG1 | syntrophin, gamma 1 | 21 | -1.13 | 0.00058 |
| LRIT2 | leucine-rich repeat, immunoglobulin-like and transmembrane domains 2 | - | -1.12 | 0.00058 |
| RPS14 | ribosomal protein S14 | - | -1.12 | 0.00058 |
| CYP46A1 | cytochrome P450, family 46, subfamily A, polypeptide 1 | 342-3p | -1.1 | 0.00058 |
| MADCAM1 | mucosal vascular addressin cell adhesion molecule 1 | hsa-let-7i | -1.1 | 0.00058 |
| MRPS26 | mitochondrial ribosomal protein S26 | 15a | -1.1 | 0.00058 |
| SLC16A8 | solute carrier family 16, member 8 (monocarboxylic acid transporter 3) | 31 | -1.1 | 0.00058 |
| ST6GALNAC5 | ST6 (alpha-N-acetyl-neuraminyl-2,3-beta-galactosyl-1,3)-N-acetylgalactosaminide alpha-2,6-sialyltransferase 5 |  | -1.18 | 0.00058 |
| EPHA10 | EPH receptor A10 | - | -1.17 | 0.00058 |
| FLJ46010 | FLJ46010 protein | - | -1.17 | 0.00058 |
| OBP2B | odorant binding protein 2B | - | -1.17 | 0.00058 |
| COL21A1 | collagen, type XXI, alpha 1 | 155 | -1.14 | 0.00058 |
| VCX | variable charge, X-linked | - | -1.14 | 0.00058 |
| NGB | Neuroglobin | - | -1.13 | 0.00058 |
| SNTG1 | syntrophin, gamma 1 | 21 | -1.13 | 0.00058 |
| LRIT2 | leucine-rich repeat, immunoglobulin-like and transmembrane domains 2 | - | -1.12 | 0.00058 |
| RPS14 | ribosomal protein S14 | - | -1.12 | 0.00058 |
| CYP46A1 | cytochrome P450, family 46, subfamily A, polypeptide 1 | 342-3p | -1.1 | 0.00058 |
| MADCAM1 | mucosal vascular addressin cell adhesion molecule 1 | hsa-let-7i | -1.1 | 0.00058 |
| MRPS26 | mitochondrial ribosomal protein S26 | 15a | -1.1 | 0.00058 |
| SLC16A8 | solute carrier family 16, member 8 (monocarboxylic acid transporter 3) | 31 | -1.1 | 0.00058 |
| ST6GALNAC5 | ST6 (alpha-N-acetyl-neuraminyl-2,3-beta-galactosyl-1,3)-N-acetylgalactosaminide alpha-2,6-sialyltransferase 5 |  | -1.18 | 0.00058 |
| EPHA10 | EPH receptor A10 | - | -1.17 | 0.00058 |
| FLJ46010 | FLJ46010 protein | - | -1.17 | 0.00058 |
| OBP2B | odorant binding protein 2B | - | -1.17 | 0.00058 |
| COL21A1 | collagen, type XXI, alpha 1 | 155 | -1.14 | 0.00058 |
| VCX | variable charge, X-linked | - | -1.14 | 0.00058 |
| NGB | Neuroglobin | - | -1.13 | 0.00058 |
| SNTG1 | syntrophin, gamma 1 | 21 | -1.13 | 0.00058 |
| LRIT2 | leucine-rich repeat, immunoglobulin-like and transmembrane domains 2 | - | -1.12 | 0.00058 |
| RPS14 | ribosomal protein S14 | - | -1.12 | 0.00058 |
| CYP46A1 | cytochrome P450, family 46, subfamily A, polypeptide 1 | 342-3p | -1.1 | 0.00058 |
| MADCAM1 | mucosal vascular addressin cell adhesion molecule 1 | hsa-let-7i | -1.1 | 0.00058 |
| MRPS26 | mitochondrial ribosomal protein S26 | 15a | -1.1 | 0.00058 |
| SLC16A8 | solute carrier family 16, member 8 (monocarboxylic acid transporter 3) | 31 | -1.1 | 0.00058 |
| ST6GALNAC5 | ST6 (alpha-N-acetyl-neuraminyl-2,3-beta-galactosyl-1,3)-N-acetylgalactosaminide alpha-2,6-sialyltransferase 5 |  | -1.18 | 0.00058 |
| EPHA10 | EPH receptor A10 | - | -1.17 | 0.00058 |
| FLJ46010 | FLJ46010 protein | - | -1.17 | 0.00058 |
| OBP2B | odorant binding protein 2B | - | -1.17 | 0.00058 |
| COL21A1 | collagen, type XXI, alpha 1 | 155 | -1.14 | 0.00058 |
| VCX | variable charge, X-linked | - | -1.14 | 0.00058 |
| NGB | Neuroglobin | - | -1.13 | 0.00058 |
| SNTG1 | syntrophin, gamma 1 | 21 | -1.13 | 0.00058 |
| LRIT2 | leucine-rich repeat, immunoglobulin-like and transmembrane domains 2 | - | -1.12 | 0.00058 |
| RPS14 | ribosomal protein S14 | - | -1.12 | 0.00058 |
