# Supplementary material for: Disease-Associated miRNA-mRNA Networks in Oral Lichen Planus
Source: PLoS One. 2013 May 27;8(5):e63015. doi: 10.1371/journal.pone.0063015 (PMC3664564; doi:10.1371/journal.pone.0063015)
Supplement: Table S2 — miRNAs, differentially expressed between OLP patients and healthy individuals. All differentially expressed non-coding RNAs associated with a characterized miRNA are listed. Fold changes are based on ratios (relative expression in lichen planus patients vs. relative expression in healthy individuals); p-values are based on a Mann-Whitney U-test. (DOCX) [file pone.0063015.s002.docx]

**Supplemental Table 2** miRNAs, differentially expressed between OLP patients and healthy individuals. All differentially expressed non-coding RNAs associated with a characterized miRNA are listed. Fold changes are based on ratios (relative expression in lichen planus patients vs. relative expression in healthy individuals); p-values are based on a Mann-Whitney U-test.

| **miRNA name** | **Sequence** | **Alignments**  [Chr.:Start-End (Strand)] | **Fold change** | **p-value** |
| --- | --- | --- | --- | --- |
| **Upregulated miRNA transcripts** | | | | |
| hsa-miR-31 | AGGCAAGAUGCUGGCAUAGCU | 9:21502114-21502184 (-) | 4.06 | 0.0111 |
| hsa-miR-146a | UGAGAACUGAAUUCCAUGGGUU | 5:159844937-159845035 (+) | 3.37 | 0.0012 |
| hsa-miR-155 | UUAAUGCUAAUCGUGAUAGGGGU | 21:25868163-25868227 (+) | 2.89 | 0.0111 |
| hsa-miR-21 | UAGCUUAUCAGACUGAUGUUGA | 17:55273409-55273480 (+) | 2.56 | 0.007 |
| hsa-miR-132 | UAACAGUCUACAGCCAUGGUCG | 17:1899952-1900052 (-) | 2.25 | 0.0379 |
| hsa-miR-146b-5p | UGAGAACUGAAUUCCAUAGGCU | 10:104186259-104186331 (+) | 2.18 | 0.007 |
| hsa-let-7i | UGAGGUAGUAGUUUGUGCUGUU | 12:61283733-61283816 (+) | 1.55 | 0.0379 |
| hsa-miR-143 | UGAGAUGAAGCACUGUAGCUC | 5:148788674-148788779 (+) | 1.40 | 0.0175 |
| hsa-miR-15a | UAGCAGCACAUAAUGGUUUGUG | 13:49521256-49521338 (-) | 1.38 | 0.0262 |
| hsa-miR-342-3p | UCUCACACAGAAAUCGCACCCGU | 14:99645745-99645843 (+) | 1.32 | 0.0379 |
| hsa-miR-425 | AAUGACACGAUCACUCCCGUUGA | 3:49032585-49032671 (-) | 1.25 | 0.0175 |
| hsa-miR-183 | UAUGGCACUGGUAGAAUUCACU | 7:129201981-129202090 (-) | 1.22 | 0.0111 |
| hsa-miR-26b | UUCAAGUAAUUCAGGAUAGGU | 2:218975613-218975689 (+) | 1.22 | 0.0379 |
| hsa-miR-335 | UCAAGAGCAAUAACGAAAAAUGU | 7:129923188-129923281 (+) | 1.19 | 0.0262 |
| **Downregulated miRNA transcripts** | | | | |
| hsa-miR-923 | GUCAGCGGAGGAAAAGAAACU | 17:30502292-30502346 (-) | -1.68 | 0.0379 |
| hsa-miR-30a | UGUAAACAUCCUCGACUGGAAG | 6:72169975-72170045 (-) | -1.13 | 0.0175 |
